# Weak Calibration of a Visible Light Positioning System Based on a Position-Sensitive Detector: Positioning Error Assessment

**DOI:** 10.3390/s21113924

**Published:** 2021-06-07

**Authors:** Álvaro De-La-Llana-Calvo, José-Luis Lázaro-Galilea, Alfredo Gardel-Vicente, David Salido-Monzú, Ignacio Bravo-Muñoz, Andreea Iamnitchi, Rubén Gil-Vera

**Affiliations:** 1 Department of Electronics, University of Alcalá, Alcalá de Henares, 28801 Madrid, Spain; alfredo.gardel@uah.es (A.G.-V.); ignacio.bravo@uah.es (I.B.-M.); andreea.iamnitchi@edu.uah.es (A.I.); ruben.gilv@edu.uah.es (R.G.-V.); 2 Institute of Geodesy and Photogrammetry, ETH Zürich, 8093 Zürich, Switzerland; david.salido@geod.baug.ethz.ch

**Keywords:** indoor positioning system (IPS), visible light positioning (VLP), position sensitive detector (PSD), soft/weak calibration, genetic algorithms

## Abstract

Reduced deployment and calibration requirements are key for scalable and cost-effective indoor positioning systems. In this work, we propose a low-complexity, weak calibration procedure for an indoor positioning system based on infrastructure lighting and a positioning-sensitive detector. The proposed calibration relies on genetic algorithms to obtain the relevant system parameters in the real positioning environment without a priori information, and requires a low number of simple measurements. The achievable performance of the proposal was assessed by direct comparison with a formal offline calibration method requiring complex dedicated infrastructure and instruments. The comparative error assessment showed that the maximum accuracy reduction compared to the significantly more costly formal calibration was below 25 mm, and the overall absolute positioning error was smaller than 35 mm with orientation errors of around 0.25°. The performance achieved with the proposed weak calibration procedure is sufficient for many indoor positioning applications and largely reduces the cost and complexity of setting up the positioning system in real environments.

## 1. Introduction

The problem of indoor localization has been the subject of intense study and research in recent years. So far, successful proposals have been developed to provide solutions for specific applications, with varying degrees of accuracy and complexity. However, universal implementation of global positioning systems for outdoor areas is still far from being achieved. The ultimate objective is to combine indoor and outdoor positioning systems. The challenge is to be able to provide an end user with a continuous and transparent navigation solution, regardless of whether one has outdoor coverage or is in an indoor environment. Knowing the user’s position can be of great added value; it can expand the capabilities of multiple applications, especially for activities in indoor environments. In indoor positioning, where the environment is complex (walls, objects, etc.), no technology prevails as global navigation satellite systems (GNSS) do in outdoor positioning systems. Sometimes the target environment restricts the design to a particular indoor positioning system (IPS) technology, directly related to accuracy, range, or scalability [1,2,3,4,5]. Indoor positioning data shall enable numerous relevant applications, such as pedestrian tracking [6]; location-based services [7,8] in public and commercial centers [9]; assistance services in daily activities (ambient assistant living (AAL)) [10]; location and tracking of users in geriatric and hospital centers [11,12]; location and tracking of emergency intervention agents (e.g., police/firefighters) [13,14,15]; location and guidance of autonomous vehicles in industrial environments and automated car parks [16,17]; tracking of high value goods during storage; extra information for users via augmented reality [18,19]; and Internet of Things (IoT) [20].

Multiple IPSs have already been deployed, such as [9,21,22,23]. As there is no prevailing technology for indoor positioning, several research efforts have used mixed indoor positioning hybrid systems based on different technologies. Many different IPSs have been proposed over the years [3,24], some of which are based on computer vision [24]; radio waves, such as ultrawideband (UWB) [25] and radio frequency identification (RFID) [26]; ultrasound [27]; optical signals [28]; and more recently, inertial measurement units (IMUs) [6,29,30] and radio frequency (RF) communications networks such as the global systems for mobile communications (GSM) and wireless local area networks (WLANs) [31]. Some technologies, such as infrared and ultrasound, are very low-cost IPS solutions that are easy to deploy, low maintenance, provide highly accurate location results, and can be used in a wide range of applications. There are many requirements and parameters to consider when making the choice of technology for a given IPS application. As [1] indicates, the following parameters can be considered: measurement accuracy, location accuracy, coverage area, required infrastructure, market maturity, data security and privacy, update speed of infrastructure nodes and mobile agents, end-user interface, system integrity, robustness, availability, scalability, number of potential users, possible degree of intrusion, legal coverage, etc. Advances in materials, electronics, and communication technologies facilitate continuous improvement in the performance of available sensing systems. The choices of sensors clearly depend on the applications’ and users’ requirements. The continuous evolution of IPSs can be shown through various works aimed at reviewing the state of the art [1,2,3,4,5].

Due to the deployment of LED lighting, more and more solutions are being developed that use visible light to both illuminate and communicate/transmit data (VLC—visible light communication) and positioning (visible light positioning) [32,33,34,35,36,37,38,39].

There are different measurement principles for positioning with VLP, including trilateration through phase measurements (PoA) [40] or received strength measurements (RSSS) [41], and triangulation [28] through angle-of-arrival (AoA) measurements. On the other hand, the effect of multipath (MP) due to different light reflections in the environment is one of the most important sources of error in VLP systems. Therefore, in [42] it was shown how AoA is more immune to MP than PoA, after modeling the multipath effect [43] with an accurate light reflection model suitable for this purpose [44].

When focusing, therefore, on the VLP based on angle of arrival (AoA) measurements, there are different alternatives depending on the types of sensors used; the most relevant are the works based on cameras [24], QADA sensors [45], photodiode arrays [46], and PSDs [47]. Each of the alternatives has its advantages and disadvantages. Broadly speaking, cameras achieve high measurement accuracy, but require high processing power, and the measurement refresh rate is low. QADA sensors such as photodiode arrays have the advantage that the processing is usually less burdensome; they also have larger bandwidths than other sensors, but need several emitters in the case of QADA or several photodiodes in the case of arrays for positioning. PSD-based systems have high accuracy and can achieve higher refresh rates than cameras, but have smaller bandwidths than QADA sensors and photodiodes. Depending on the application requirements, one alternative or the other can be used.

When AoA is used as a measurement technique, it is necessary to use a lens system or a tiny aperture (pinhole). In the case of photodiode arrays, the use of such systems is not necessary, since they have a different configuration. They obtain the AoA not by the sensor itself but by the signal strength received from numerous photodiodes placed in a certain configuration. When using lenses it is necessary to know with high precision the intrinsic parameters of the sensors, plus the lens assembly and the distortion parameters. The precision of the positioning will depend directly on the errors made in the determination of these parameters. This is why there are numerous calibration methods designed primarily for the calibration of [48] cameras. These are based fundamentally on acquiring pictures of a checkerboard and from the corners of the different squares to obtain the distortions of the system.

We focused our research on PSD-based VLP, and since for PSDs it is necessary to attach lenses to obtain the AoA, calibration had to be performed as well. In [49] a calibration process based on Zhang’s work [48] but adapted for PSDs was presented. In this case, checkerboards were no longer used, since the PSD did not form an image; instead, a template was used with multiple led emitters placed at known points on a surface, similarly to placing them on the corners of checkerboard squares. It was shown that this calibration method obtains quite good results, but it has some drawbacks, many of them inherited from camera calibration systems. First of all, let us remark that it is a long and laborious process. The calibration template has to be placed in multiple positions at different angles, covering most of the PSD’s range. Since there must be many LEDs, in our case 16, and to avoid possible interference, each one of them emits the light sequentially, to acquire a single “image” it is necessary to spend approximately 2 min. Another problem can be found when placing and soldering the LED emitters, as this manufacturing process will always produce small errors. Usually, this error is greater than the error made by a printer when printing a checkerboard. For this reason, it will be necessary to take more “images” to reduce this error as much as possible. Another very important aspect is that once the calibration has been carried out, any small maladjustment, for example, in the handling or in the final installation, means that the calibration process will have to be repeated again. To simplify the process, in this paper we propose a weak calibration method.

The paper is organized as follows: First an overview of previous research underlying this work is provided in Section 2. In Section 3, the proposal of weak calibration using the genetic algorithm is described. Section 4 describes the setup of the experimental evaluation and the computation of system parameters. In addition, the results are compared with those of a formal calibration, and the dependence of the results on the points chosen is studied. Finally, conclusions are summarized in Section 5.

## 2. Background

In this section we present some recent research on the VLP topic related to this work, both from our research group and others.

In previous works carried out by our research group, the influence of the multipath effect on IPSs based on optical signals has been analyzed. In [44] a light reflection model was developed to characterize how light will be reflected by different materials. Thanks to this reflection model, an algorithm was developed [43] which, given a certain environment, allows one to obtain the signal that would be received by the receivers of the positioning system. With this algorithm, in [42] it was demonstrated that the AoA measurement technique is more immune to the multipath effect than the PoA technique. AoA-based systems are affected by different electronic component tolerances and drifts. Therefore, an electrical calibration method was developed in [50] to compensate for these effects.

In [51], it is shown how the frequency division multiple access (FDMA) technique is the most suitable for IPSs based on PSDs. FDMA allows one to discriminate between the different emitters of the system with almost no interference, unlike code division multiple access (CDMA), which due to multiple access interference (MAI), causes errors when obtaining the point of impact. In [52] is shown the implementation of an IPS based on PSDs [51] in a microcontroller unit (MCU)-based system-on-chip (SoC) system. In [52] it is demonstrated that it is possible to integrate into an MCU both the hardware and software requirement for signal acquisition and processing, for an IPS detector.

Recent research by other groups is presented below.

In [53], the actual communication characteristics of a VLP system that uses a QADA receiver are studied. The authors calculated the signal-to-noise ratio and bit-error-rates for a range of scenarios, proving that communication will not be a limiting factor when using QADA in VLP systems.

In [54] is presented an autonomous method of collecting data for VLP, and a comprehensive investigation of VLP using a large set of experimental data is presented. RSS data were collected using a method that utilizes consumer grade virtual reality (VR) tracking for accurate ground truth recording. The quality and volume of the data allow for robust study of machine learning (ML)- and channel model-based positioning utilizing visible light.

A sensor utilizing low-range infrared (IR) signals in the line-of-sight (LOS) context, providing high precision AoA estimation, is presented in [46]. The proposed sensor was used as a pragmatic solution to the localization problem that avoids NLOS propagation issues by exploiting the powerful concept of the wireless sensor network (WSN). The evaluation outcomes reached centimeter-level accuracy.

In [55] a three-dimensional VLP algorithm using the Cayley–Menger determinant (CMD) with a cost function was proposed, and it was experimentally tested to track a drone for industrial applications. The proposed algorithm uses optical RSS for estimating the drone’s 3D position without prior knowledge of its height.

Ref. [56] proposed an artificial neural network (ANN)-based approach for accurate modeling and positioning with on-site data. Likewise, the proposed approach was proven applicable to accurate modeling of initial time delay distributions of LED chips in VLP systems based on phase differences of arrival (PDOA). To improve the robustness by mitigating the impact of intensity variations, they introduced a selection strategy utilizing both PDOA and RSS measurements.

In [57], a fast and high-accuracy single-LED based VLP system was proposed. Firstly, an unbalanced single-LED VLP algorithm was proposed to increase the positioning accuracy and reduce the computational complexity. Secondly, a fast beacon searching algorithm was proposed to further reduce the processing time for each captured image.

A new optical signal modulation technique suitable for intensity-modulation/direct-detection (IM/DD)-based VLP systems was developed in [58]. The comparative simulations reported that the proposed scheme improved the signal-to-noise ratio (SNR) by more than 4.6 dB, reduced the channel error by 3.5 times or more, and reduced the peak-to-average power ratio (PAPR) by more than 6 dB, excluding time division multiple access (TDMA).

According with the introduction and considering the background of the existing research on the topic, the work presented here focuses the research on VLP systems based on PSDs. This work is based on the work shown in [47]. In that work, several proposals were made for LPS based on optical signals from PSDs as a function of the number of emitters within the field of view of the emitter. Experimental tests were performed in a real environment. Total stations (TS) were used to obtain accurate ground truth. The accuracy and precision of the each proposal were evaluated. To obtain these results, a formal geometric calibration had to be performed beforehand, as shown in [49]. This calibration requires taking different images of a very precise template, at different positions and angles. With the information retrieved from several images (usually more than 10), the parameters of the PSD + lens system, such as focal length, optical center, and radial and tangential distortion parameters, can be obtained. It is a long and laborious process. Besides, any small movement of the lens, for example, moving it from the calibration bench to its position on the mobile agent, modifies the parameters, and it is necessary to do the process again.

This paper proposes a weak calibration procedure to obtain some parameters of the PSD + lens system that allow one to obtain the positioning in an accurate way. A comparison between the positioning results obtained with the proposed model and formal calibration is presented. An experimental evaluation in a real environment is provided to show the benefits of the proposed method.

The main contributions of this work are:

- The calibration proposal is a quick method of calibration compared to formal calibration. System parameters can be obtained with only six measurements.
- Calibration is performed in the actual environment, so no complex infrastructure is required for receiver calibration.
- The absolute positioning error obtained using the proposed calibration is below 35 mm, so the accuracy achieved is higher than what is achievable for most other existing IPS alternatives.
- Highly qualified experts are not needed to calibrate the modules and put them into operation almost immediately.

## 3. Identification of IPS Parameters Using Genetic Algorithms

To position a mobile agent in a plane, when using AoA in a PSD-based positioning system, it is necessary to receive the signal from at least two emitters. The computation requires one to know the height of the motion plane to obtain the rotation of the mobile agent. It is also possible to obtain the position from the signal of only one emitter if the rotation of the agent is already known by means of external methods or sensors. In [47], the positioning proposals using 1, 2, and 3 or more emitters are shown in detail. This last case allows one to obtain the total pose, i.e., 3D coordinates and three angles that define its orientation.

**Figure 1:**
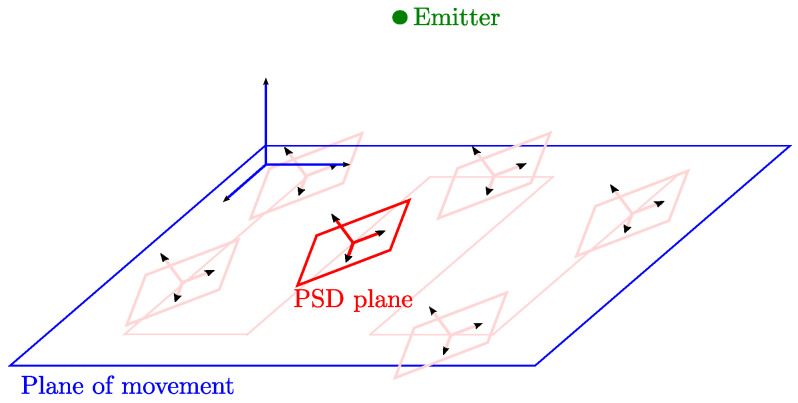
Relative position between the plane of movement and the plane of the PSD.

Focusing in the positioning using one or two emitters, one or more of the following conditions may occur in real environments:

- The normal vector of the PSD surface is not parallel to the normal vector of the motion plane. This is usually due to errors in the placement of the sensor holder. An example is shown in Figure 1 showing the relative situation between the motion plane and the sensor plane. This difference in orientation can be defined with the Euler angles. From these angles, we obtain the R matrix of rotation between one plane and the other.
- The parameters focal length *f* and center of projection of the PSD + lens $\left(C_{x},C_{y}\right)$ are unknown, along with the radial and tangential distortion parameters. These parameters are those that would be obtained with a formal calibration and are essential to be able to position by triangulation with AoA measurements.
- The emitter position is not known with sufficient accuracy within the environment $\left(X_{e},Y_{e},Z_{e}\right)$. When placing emitters on the ceiling, the required accurate measurement tools are not always available to obtain precise positioning values.

Since it is not always possible to know all the parameters of a system with the required precision, our aim is to propose a weak calibration process that allows, with few measurements from the real scenario, obtaining the parameters needed calculate the position of the PSD accurately. It is worth noting that the more system parameters one knows, the fewer there are to estimate, and the better the results can be expected to be.

**Figure 2:**
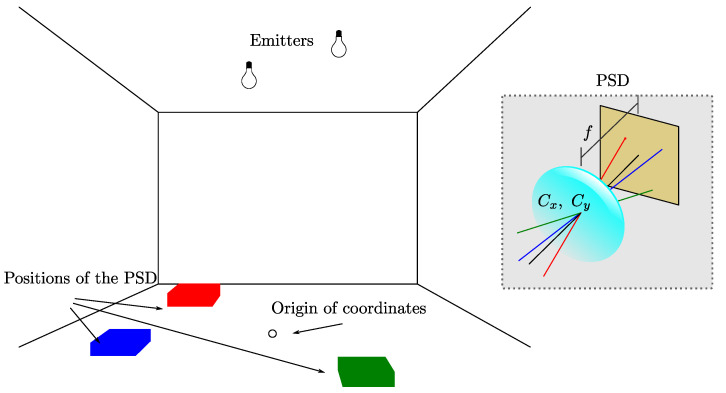
Calibration process.

The steps proposed for the calibration process are listed below. Figure 2 helps with following the process.

- Choose a reference origin for the coordinate system of the environment.
- Place the mobile agents with the PSDs at different known positions in the environment along the motion plane, maintaining $Z=Z_{r}$. Each position is defined as $\left(X_{r}^{n},\;Y_{r}^{n},\;Z_{r}^{n}\right)$, where *n* is the receiver position number out of a total of *N* distinct positions.
- Obtain, for each position, the incident point on the PSD surface, $\left(x^{jn},\;y^{jn}\right)$ from one or more emitters *J*, where *n* defines the position of the receiver and *j* defines the incident point of the *j*th emitter.
- Obtain the system parameters that minimize the reprojection error by means of an optimization and fitting process, such as a genetic algorithm.

In this work, the genetic algorithm (GA) [59,60,61] is used because it presents some advantages over other optimization techniques, including the following:

- GAs are less likely to converge on local maxima or minima.
- They are simple to implement, since the only information needed is the objective function and the corresponding constraints.
- GAs can be implemented in distributed or parallel implementations.
- Genetic algorithms are appropriate for optimizing non-differentiable functions or functions profuse with local minima, since GAs are global search methods that do not employ gradient information.

Next, we summarize how the genetic algorithm works, as related in [62].

- The algorithm starts by creating a random initial population.
- Next, the algorithm creates a sequence of new populations. At each step, the algorithm uses the individuals from the current generation to create the next population. To create the new population, the algorithm performs the following steps
  - It scores each member of the current population by calculating its fitness value. These values are called raw fitness scores.
  - It scales the raw fitness scores to convert them into a more usable range of values. These scaled values are called expected values.
  - It selects members, called parents, based on their probabilities.
  - Some of the individuals in the current population who have great fitness are chosen as the elite. These elite individuals pass values on to the next population.
  - It produces children from the parents. Children are produced by making random changes in a single parent—mutations—or by combining vector entries from a pair of parents—crossing.
  - It replaces the current population with offspring to form the next generation.
- The algorithm stops when one of the stopping criteria is met.

**Figure 3:**
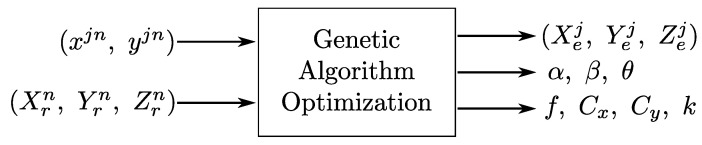
Genetic algorithm used to estimate the IPS parameters.

Figure 3 shows the inputs and outputs of the genetic algorithm. The inputs of the genetic algorithm are the PSD surface incident points $\left(x^{jn},\;y^{jn}\right)$ and the corresponding 3D positions of the PSD in the environment with respect to the chosen coordinate origin $\left(X_{r}^{n},\;Y_{r}^{n},\;Z_{r}^{n}\right)$.

The output data of the genetic algorithm are the parameter values: position of the *j*th emitter in the environment $\left(X_{e}^{j},\;Y_{e}^{j},\;Z_{e}^{j}\right)$, misalignment angles of the PSD system with respect to the plane of movement (α,β,γ), the intrinsic parameters $\left(f,\;C_{x},\;C_{y}\right)$, and the lens distortion parameter (k).

The variable to be minimized with the genetic algorithms is the average distance error between the measured impact points and the projections of the impact points obtained analytically with the parameters of the system to be optimized.

To obtain the distance error value, we start from the pinhole model, whose expression is shown in (1).

(1) $$(\begin{array}{c} sx^{jn^{\prime }}\\ sy^{jn^{\prime }}\\ s \end{array})=\underset{A}{\underset{︸}{\left(\begin{array}{ccc} f & 0 & C_{x}\\ 0 & f & C_{y}\\ 0 & 0 & 1 \end{array}\right)}}\underset{R}{\underset{︸}{\left(\begin{array}{ccc} r_{11} & r_{12} & r_{13}\\ r_{21} & r_{22} & r_{23}\\ r_{31} & r_{32} & r_{33} \end{array}\right)}}\left(\begin{array}{c} X_{e}^{j}-X_{r}^{n}\\ Y_{e}^{j}-Y_{r}^{n}\\ Z_{e}^{j}-Z_{r}^{n} \end{array}\right)$$

where the rotation matrix R is obtained from the angles α,β,θ. $\left(x^{jn^{\prime }},\;y^{jn^{\prime }}\right)$ is the projection of the *j*th emitter onto the sensor surface of the *n*th receiver position, calculated with the parameters to be optimized $\left(X_{e}^{j},\;Y_{e}^{j},\;Z_{e}^{j}\right),\;\alpha ,\;\beta ,\;\gamma ,\;f,\;C_{x},\;C_{y}$. Therefore, $\left(x^{jn^{\prime }},\;y^{jn^{\prime }}\right)$ is the point of impact in the absence of distortion.

Additionally, it is necessary to correct the distortion of the measured impact points $\left(x^{jn},\;y^{jn}\right)$. In general there are two types of distortion, radial and tangential distortion. We have considered only the radial distortion, since the tangential distortion will be corrected to some extent by estimating the misalignment of the normal vectors for the sensing plane and the motion plane. It must be taken into account that this weak calibration is computed with a few number of incidence points, so that increasing the number of parameters to be optimized in excess could lead the algorithm to converge to a non-optimal solution. The radial distortion is modeled according to:

(2) $$d_{x}^{jn}=\left(x^{jn}-C_{x}\right)\left(k_{1}r_{jn}^{2}+\ldots +k_{m}r_{jn}^{2m}\right)$$

(3) $$d_{y}^{jn}=\left(y^{jn}-C_{y}\right)\left(k_{1}r_{jn}^{2}+\ldots +k_{m}r_{jn}^{2m}\right)$$

where $r_{jn}$ is the Euclidean distance between the measured point of impact $\left(x^{jn},\;y^{jn}\right)$ and $k_{i=1,\ldots ,m}$ are the *m* parameters that model the lens radial distortion.

Distortion is corrected according to (4) and (5), thereby obtaining the coordinates of the corrected impact point $\left(x^{jn^{\prime \prime }},\;y^{jn^{\prime \prime }}\right)$.

(4) $$x^{jn^{\prime \prime }}=x^{jn}-d_{x}^{jn}$$

(5) $$y^{jn^{\prime \prime }}=y^{jn}-d_{y}^{jn}$$

As already mentioned, the goal is to obtain the values of the parameters that minimize the error value obtained as the sum of the Euclidean distances between the coordinates of the reprojection $\left(x_{i}^{jn^{\prime }},\;y_{i}^{jn^{\prime }}\right)$ and the coordinates measured after correction of the distortion $\left(x^{jn^{\prime \prime }},\;y^{jn^{\prime \prime }}\right)$, according to:

(6) $$\frac{1}{NJ}\sum_{n=1}^{N}\sum_{j=1}^{J}\sqrt{{\left(x^{jn^{\prime }}-x^{jn^{\prime \prime }}\right)}^{2}+{\left(y^{jn^{\prime }}-y^{jn^{\prime \prime }}\right)}^{2}}$$

Depending on the number of unknown parameters, it will be necessary to take more or less points for the calibration process. The points should be taken so as to cover most of the field of view of the receiver. If the distortion is large, and the distortion parameters are not known, the number of points needed for calibration will be high.

In general, there will be an approximate knowledge of the value of the output parameters of the genetic algorithm, which allows one to restrict the range of values it can take, and therefore, to ensure that an optimal solution with values close to the real values of the parameters will be obtained. For example, it is possible to produce an approximation of the 3D coordinates of the emitters, measuring them with a certain amount of error; the focal length, obtained from the lens datasheet; or for example, to consider that the plane of the ceiling (emitters) and the plane of the ground (receiver) are going to be coplanar with errors of only a few degrees.

## 4. Results

This section shows the results obtained when the proposed calibration process is applied. The results and the comparison with the formal calibration considering that the sensor receives the signal from two emitters are shown.

### 4.1. Experimental Setup

**Figure 4:**
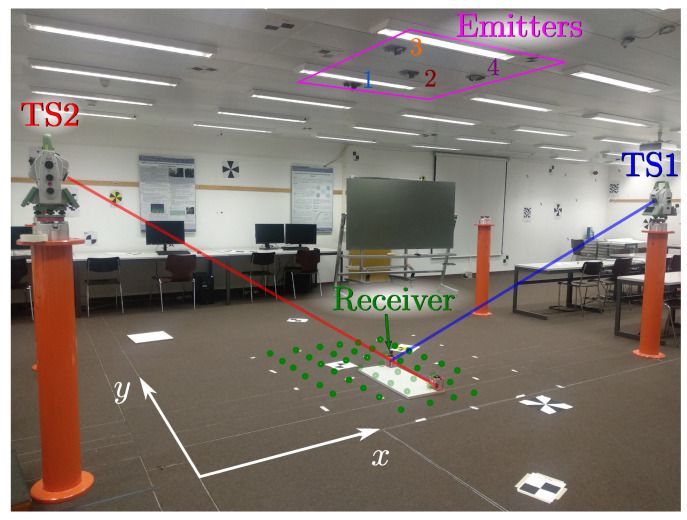
Test environment.

The setup is described in detail in [47]. Figure 4 shows a picture of the test environment. As a summary, the main features are listed below:

- The emitters were located on the ceiling. There were four emitters, depending on the tests to be performed, the signals from two different emitters were processed. The identifier and the modulation frequency of each emitter are shown in Table 1.
- The receiver was placed at different points on the floor.
- Two total stations were used to obtain, with sub-millimeter precision, the ground-truth positioning of the receiver and its rotation.

**Figure 5:**
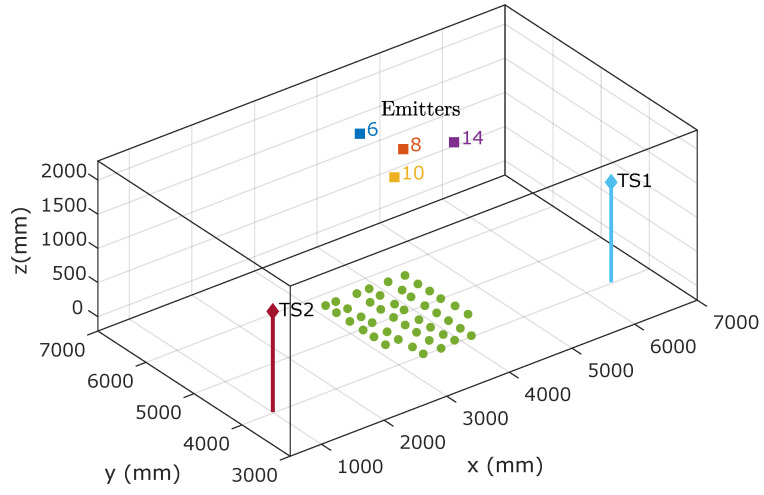
Positions of the emitters, receivers (green), and total stations in the test environment.

Figure 5 shows the positions of the receiver (green points) and the emitters within the environment, along with the positions of the total stations used to obtain the ground truth values.

### 4.2. System Parameters Computation

**Figure 6:**
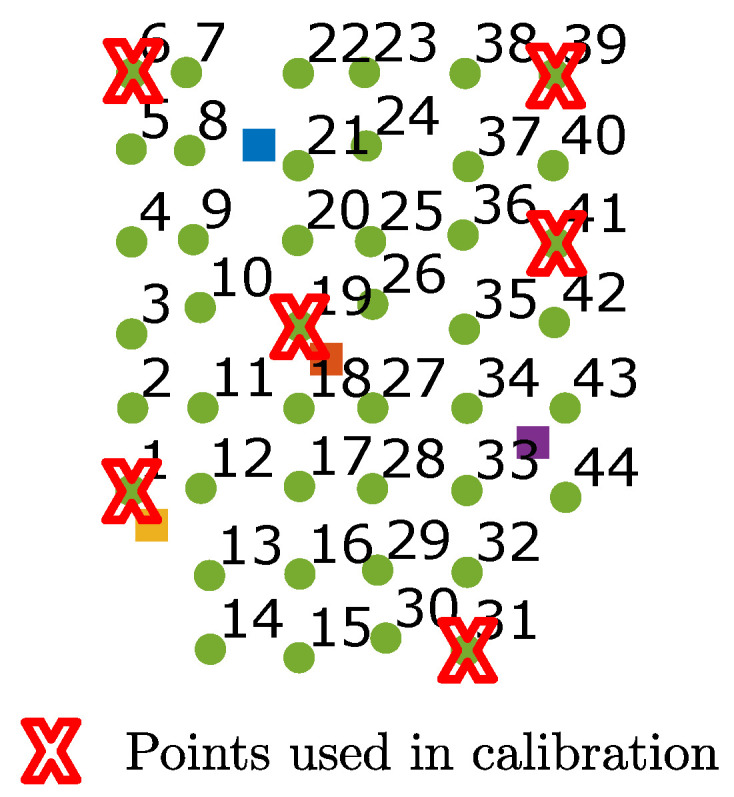
Points used in the calibration process.

To perform the calibration, the receiver was placed in a total of six positions in the coverage area of the two emitters. In this case, in each position the signal impact points of emitters 2 and 4 were obtained. The receiver positions used to obtain the parameters are shown in Figure 6. The points were chosen to try to cover as much of the area as possible (in the corners) and by placing some points near the center. A total of six points was chosen as a trade-off between using minimal points to calibrate (faster calibration process) and obtaining an acceptable solution. The number of parameters to obtain was 13 (six coordinates of the two emitters, plus the three Euler angles, plus the three intrinsic parameters and the distortion parameter). Using six receiver positions, we got 12 values of $\left(x_{i}\right)$ and 12 $\left(y_{i}\right)$ (using two emitters there are two times more impact points than receiver positions), so we used 24 equations to obtain 13 parameters.

To know the receiver positions used in the calibration, the ground truth data obtained by the total stations were used. To emulate a real measurement in a real environment where total stations are not available, a Gaussian random error with a standard deviation of 5 mm (value obtained experimentally after several measurements by usual measuring instruments compared to the total station) was added to these positions.

It was also mandated that the receiver always moved in the same plane. To calibrate, it is necessary to know the rotation of the receiver at each point, and when measuring in a real environment there can always be small errors when positioning the receiver. For this reason, in the calibration process we fixed the rotation, with a value of −90°, when, in fact, the angles measured by the TS vary from −88° to −92° (which means that we had errors of ±2° in the measurements that the calibration method must tolerate).

To consider that possibly the PSD is not coplanar with the plane of movement (Figure 1), the calibration process had to obtain the Euler angles α, β, and γ to correct those possible orientation errors, which were fixed in all positions. The range of values that these angles can take was limited to ±2°, since we estimated that this would be the maximum error that we would have between the two planes.

The calibration procedure provides the coordinates of the emitters. A range of possible values has been set for the emitter coordinates with respect to those measured with the total station of ±2 cm. This value was chosen because after performing some manual measurements and comparing them with the real values, the maximum error obtained when measuring the emitter coordinates manually was ±2 cm. This value is higher than the one established in the receiver coordinates, as manual measurement of the ceiling increases the errors. The calibration process was set up so that the *z* coordinates of the emitters were the same.

Finally, the calibration returned the parameters of the system PSD + lens, such as focal length *f*, optical center $C_{x}$ and $C_{y}$, and radial distortion parameter *k*.

In summary, the input and output values of the calibration method for this specific case were:

- The inputs:
  - The coordinates of the six different positions of the receiver (Figure 6) with a deviation error of 5 mm in the receiver position (to emulate manual positioning).
  - For each receiver position, the impact points obtained from the signals of two emitters (number 2 and number 4) have been used.
  - It was defined that the rotation of the mobile agent remained fixed at −90° at all positions (with an error of up to ±2 degrees).
- The restrictions:
  - The range of values of the emitter coordinates was ±2 cm with respect to the measured value.
  - The range of values that the Euler angles could take was limited to ±2°.
- The outputs:
  - Coordinates of the two emitters.
  - The Euler angles of the sensor rotation with respect to the plane.
  - Parameters of the system PSD + lens, *f*, $C_{x}$, and $C_{y}$; and radial distortion parameter *k*.

To clarify the coordinates of the emitters, in despite of being an output of the genetic algorithm, it was necessary to introduce their approximate coordinates measured with usual measuring instruments beforehand, hence the error ±2 cm.

In this case, the function ga of Matlab that finds the minimum of a function using the genetic algorithm [63,64] was used to obtain the values of the system parameters.

The values of the outputs obtained from the genetic algorithm, although not shown because they are results of an abstract model, were those used to obtain the positioning of the receiver.

### 4.3. Comparison with Formal Calibration

Once the parameters were obtained from the genetic algorithm, with the incidence points from the two emitters, the positions of the sensors were calculated. We proceeded in a similar way to [47] in order to compare our method with formal calibration.

When receiving signals from two emitters, it is possible to know the angles and heights along the plane of the emitters and the plane of movement of the receiver. If these heights are obtained externally with high accuracy, or as in this case, we know them because we have performed the calibration, the positioning results can be improved with respect to cases where the height is calculated in the positioning process. Therefore, both cases will be analyzed: when the height is calculated and when the height is known.

Besides, it should be noted that in this section we focus on the accuracy of the position measurement, i.e., only taking into account systematic errors. For this purpose, a high integration time has been used in order to reduce random errors as much as possible. The integration time was configured at 15 s, as it was experimentally demonstrated to provide sufficient absorption of random variations. The study of the accuracy due to random errors was performed in [47]. Since errors due to miscalibration affect only to the measurement accuracy, there is no point in analyzing the precision in this work.

**Figure 7:**
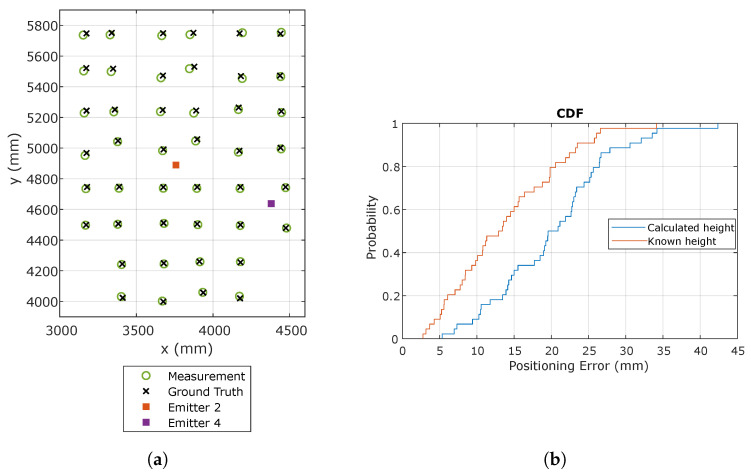
Positioning results using emitters 2 and 4. (**a**) Estimated and true receiver positions using calculated height; (**b**) CDF of the positioning error.

Figure 7 shows the calculated positions and the true positions along with the cumulative distribution function (CDF) of the positioning error, measured as the Euclidean distance between the actual point and the calculated point.

**Figure 8:**
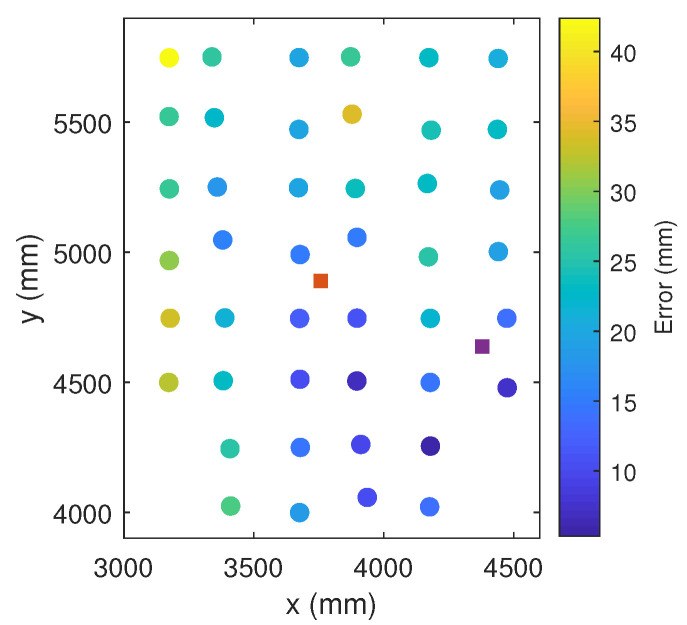
Receiver positioning errors shown graphically by calculating the height.

Figure 8 shows graphically the positioning error in different colors as a function of the error on the receiver coordinates.

It can be seen how the errors increase slightly as the receiver moves away from the emitter positions. This effect may be due to the calibration process, which only considers one distortion parameter: the further the receiver moves away from the emitters, the less distortion is well-corrected.

As we used four emitters (Figure 5), there were different alternatives to choosing two pairs of emitters. In this work, besides this first case considering one emitter in the center and one in the corner, the same procedure was performed with a different configuration—two emitters placed in the corners. With these two cases we cover practically all the possibilities, since due to the symmetry of the setup, the results of other possible configurations would be similar.

Table 2 shows the mean, standard deviation, and maximum values of the positioning errors of both case studies. Table 3 shows the same values for a formal calibration obtained by following the procedure given in [47].

It can be seen that the position errors in both cases are low and not very different from those obtained by formal calibration procedure. Mean errors are about 6–13 mm higher when using the genetic algorithms calibration procedure.

The accuracy measurement results for the rotation angle are shown similarly to in [47]. To obtain the values, the receiver was located under emitter 2 and was rotated by a total of 34 degrees.

**Figure 9:**
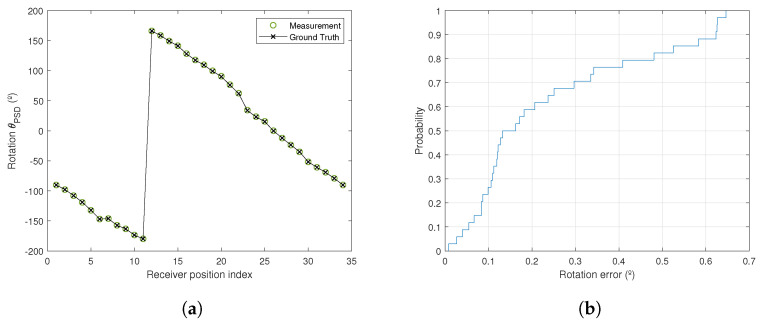
Rotation angle results using emitters 1 and 4. (**a**) Calculation of the rotation angle; (**b**) CDF of the rotation error.

The mean, standard deviation, and maximum error in the determination of the rotation angles are detailed in Table 4. Figure 9 shows the calculated rotation angles and the true rotation angles along with the cumulative distribution function (CDF) of the rotation angle error. As can be seen, the error in the measurement of the rotation was below 0.5° in 80% of cases, with an average value of 0.241°. Errors are also shown for formal calibration was performed.

It can be seen that when using the weak calibration, the errors increased slightly; even so the average error was under 0.25°.

Up to this point, the results shown have been those obtained after a single run. Since a random error was introduced in the receiver position, the results are particular to that particular execution. To avoid this situation, the results shown are for 100 runs. Table 5 shows the average and SD values for the 100 runs with the average positioning error of the 44 receiver positions for the two combinations of emitters, for calculated or known height. Similarly, in Table 6, the same data are shown for the standard deviations of the positioning error of the 44 positions. In addition, three cases are shows in detail:

- Case 1. Same case as shown above. The error in the positioning of the receiver is a random error with a standard deviation of 5 mm and the error in the positioning of the emitters is limited to ±2 cm.
- Case 2. The error in the positioning of the receiver is a random error with a standard deviation of 10 mm, and the error in the positioning of the emitter is limited to ±3 cm.
- Case 3. The error in the positioning of the receiver is a random error with a standard deviation of 20 mm, and the error in the positioning of the emitter is limited to ±4 cm.

It can be seen how the results shown are higher than those shown in the Table 2, because the calibration was performed with lower error values (random errors). Even so, the average errors were below 33 mm when the height was calculated and below 16 mm when the height was known. It can be seen that even when emulating a very bad receiver position measurement, case 3, the errors were below 45 mm and below 24 mm for calculated and known height, respectively.

### 4.4. Dependence of the Results on the Points Chosen

The choices of positions for receivers will impact the final results of a calibration. It can be seen that our results are good, with an average error of less than 45 mm in the worst case, using only six receiver positions. It should be noted that equally or more importantly than the number of points used is the positions of those points. In general, the following recommendations should be followed to obtain optimal results:

- Choose the points distributed over the coverage area of the emitters.
- Do not choose points very close to the boundary of coverage for any of the emitters, since at those points the distortion of both the lens and the PSD itself makes those measurements have greater error than the rest.

To test the influence of the choice of the points, several combinations of six emitters have been analyzed, while always following the discovered criteria. The results were analyzed for case 1 without knowing the height, and the results were practically the same, with differences of less than 10% compared to those obtained in the Table 5 and Table 6.

Another factor that can influence the calibration result is the number of points chosen. As a general rule, the more points chosen, provided they are chosen appropriately, the better the results. A test was carried out in which nine points were chosen, evenly distributed in three rows and three columns out of the 44 available. In this test, case 1 was analyzed a total of 100 times, obtaining the values shown in Table 7. The mean and SD values of the mean errors of the total of the 100 emulations performed are shown.

When comparing the results of the Table 5 and Table 6 with the results of Table 7, it can be observed that as the number of points used in the calibration increased, the results improved. Specifically, there was a 5 mm improvement in the mean error.

The choice of the number of points to perform the calibration is a trade-off: the more points used, the better results can be expected to be, but the time to acquire the signals will also increase. Additionally, note that depending on the system configuration, there will be a point at which increasing the number of points in the calibration does not appreciably improve the results.

In this work, we have mostly shown the results when using six points, as we considered that it is a good trade-off solution since similar results to the formal calibration were obtained using a fraction of the time and resources.

## 5. Conclusions

A weak calibration method has been developed to improve the position and orientation accuracy of an indoor positioning system based on a PSD performing AoA measurements from modulated infrastructure lighting. The proposed calibration relies on genetic algorithms to estimate the calibration constants without a priori knowledge of the system parameters and requires only six measurement points within the positioning environment.

The positioning errors using the proposed calibration process are not significantly degraded (below 2 cm) when compared to those obtained from a far more complex and costly formal calibration. The main specific contributions and features of the proposed weak calibration are:

- The parameters required for correcting the lens distortion with sufficient accuracy can be obtained using only observations on six different positions. This prevents the need for a long and complex formal calibration process that is normally carried out in a measurement bench, thereby increasing the risk of misalignments during deployment. Conversely, the simplified model is unable to account for large lens distortions when the emitters are significantly far away from the sensor.
- Small misalignments in the orientation of the PSD with respect to the plane of motion are automatically accounted for.
- The proposed procedure is robust against inaccuracies in the ground-truth data, yielding relatively low global positioning errors despite small but non-negligible errors in the reference positions.
- The calibration procedure enables positioning with only approximated information about the position of the emitters.

The evaluation of the positioning system’s performance using the proposed weak calibration took place by comparison with the results obtained for the same system following an established calibration procedure. The degradation of accuracy was reasonably minor and largely compensated for by the reduction of calibration complexity and cost. The performance is sufficient for a wide range of indoor positioning applications whose overall cost and scalability should be enhanced by the simplified calibration method proposed herein.

## Figures and Tables

**Table 1:** Index and frequency modulation of the emitters.

**Index of Emitter**	1	2	3	4
**Modulation Frequency (kHz)**	6	8	10	14

**Table 2:** Positioning errors using two emitters obtained by a weak calibration procedure.

	Error Using Calculated Height			Error Using Known Height		
**Indexes of the Emitters**	**Mean**	**Std**	**Max**	**Mean**	**Std**	**Max**
2–4	20.2 mm	7.9 mm	42.4 mm	13.7 mm	7.5 mm	34.1 mm
3–4	24.9 mm	11.6 mm	54.1 mm	8.6 mm	6.5 mm	26.9 mm

**Table 3:** Positioning errors using two emitters obtained by formal calibration.

	Error Using Calculated Height			Error Using Known Height		
**Indexes of the Emitters**	**Mean**	**Std**	**Max**	**Mean**	**Std**	**Max**
2–4	15.1 mm	6.3 mm	31.3 mm	8.9 mm	5.8 mm	27.1 mm
3–4	11.8 mm	6.3 mm	27.7 mm	9.2 mm	5.2 mm	25.1 mm

**Table 4:** Errors in the calculation of the rotation angle using emitters 1 and 4.

	Mean Error	Std Deviation Error	Maximum Error
Soft calibration	0.241°	0.202°	0.646°
Formal calibration	0.157°	0.146°	0.440°

**Table 5:** Mean error of the 44 receiver positions using calculated and known height in the three test cases.

	Error Using Calculated Height (mm)						Error Using Known Height (mm)					
**Indexes of the Emitters**	**Case 1**		**Case 2**		**Case 3**		**Case 1**		**Case 2**		**Case 3**	
	**Mean**	**std**	**Mean**	**std**	**Mean**	**std**	**Mean**	**std**	**Mean**	**std**	**Mean**	**std**
2–4	32.3	12.1	35.3	11.4	44.2	16.8	12.5	2.5	16.8	4.7	23.6	8.5
3–4	32.5	8	35.8	8.8	41.7	12.5	15.3	3.4	17.9	5.5	23.7	8.3

**Table 6:** Std error of the 44 receiver positions using calculated and known height in the three test cases.

	Error Using Calculated Height (mm)						Error Using Known Height (mm)					
**Indexes of the Emitters**	**Case 1**		**Case 2**		**Case 3**		**Case 1**		**Case 2**		**Case 3**	
	**Mean**	**std**	**Mean**	**std**	**Mean**	**std**	**Mean**	**std**	**Mean**	**std**	**Mean**	**std**
2–4	18.8	9.9	18.2	8.2	20.3	10.9	7.4	1.6	8.6	2.3	11.1	3.6
3–4	17.4	6	18.7	5.7	19.1	6.8	10.2	2.5	11.1	3.7	12.6	4.8

**Table 7:** Mean and error of the 44 receiver positions using calculated and known height and nine calibration points in the case 1.

	Error Using Calculated Height				Error Using Known Height			
**Indexes of the Emitters**	**Mean Error**		**Std Error**		**Mean Error**		**Std Error**	
	**Mean**	**std**	**Mean**	**std**	**Mean**	**std**	**Mean**	**std**
2–4	27.7 mm	7.1 mm	16.8 mm	6.5 mm	11.1 mm	2.7 mm	6.6 mm	1.2 mm
